# Prediction of Rotator Cuff Injury Associated with Acromial Morphology: A Three‐Dimensional Measurement Study

**DOI:** 10.1111/os.12774

**Published:** 2020-11-16

**Authors:** Yi‐Ming Zeng, Chen Xu, Kai Zhang, De‐Gang Yu, Jun Zhang

**Affiliations:** ^1^ Shanghai Key Laboratory of Orthopaedic Implants, Department of Orthopedic Surgery, Shanghai Ninth's People's Hospital Shanghai Jiao Tong University School of Medicine Shanghai China

**Keywords:** Acromial morphology, Rotator cuff tear, Three‐dimensional measurement, Arthroscopy

## Abstract

**Objective:**

To analyze the relationship between the acromial morphology and the related rotator cuff injury using a three‐dimensional (3D) measurement technology.

**Methods:**

For the present study, 226 patients (113 men and 113 women) who underwent shoulder Coarthroscopy from June 2015 to December 2019 at the Department of Orthopedics at our hospital were selected retrospectively. A total of 113 shoulder joints of age‐matched healthy people were selected as the control group. A 3D model coordinate system of the shoulder was established based on CT scan images. Patients were grouped according to the condition of the rotator cuff injury during surgery. The patients whose rotator cuff tear site corresponded to the 3D osseous proliferative structure of the acromion were classified into the impingement injury group (II group). The other patients were classified into the non‐impingement injury group (NII group). The acromiohumeral interval (AHI), the acromial anterior protrusion (AAP), the acromial inferior protrusion (AIP), the acromioclavicular angle (AC angle), the distance from the most medial edge of the acromial anterolateral protrusion (AALP) to the most lateral point of acromion (MLPA) (a), the distance from the most posteromedial edge of the AALP to the MLPA (b), the anteroposterior diameters of the AALP (c), and the proportion of anteroposterior diameters of AALP to the anteroposterior diameters of acromion, (c/c + d) × 100(%), were measured using the 3D shoulder model.

**Results:**

The results of the intraobserver (<5%) and interobserver variability (>87%) analysis found the parameters to have high intraobserver and interobserver concordance. There were no significant differences in age among the control group, the NII group, and the II group (*P* = 0.8416). There were significant differences in AAP among the three groups (*P* = 0.0374). The results were the same for men and women, respectively. The AAP in the control group and the NII group did not show a difference, while the AAP in the II group was increased by 26.9% (*P* = 0.015) and 25% (*P* = 0.023), respectively, compared with the NII group and the control group. AHI, AIP, and AC angles did not show significant differences among the three groups (*P* > 0.05). The (a) and (b) of the II group were significantly larger than those of the NII group; *P*‐values were 0.0119 and 0.0003, respectively. The (a) and (b) in patients with rotator cuff injuries were larger than in the normal population (*P* < 0.05). The above results were the same for men and women. This suggested that the larger width of the AALP might cause the related rotator cuff injury. The (c/c + d) in the II group was significantly larger than those in the control and the NII groups, with *P‐v*alues of 0.0005 and 0.0021, respectively. The risk of rotator cuff injury due to subacromial impingement was increased when the maximum width of the medial–lateral edge of the AALP exceeded 16.8 mm (17.4 mm in men, 15.1 mm in women), the maximum width of the posterior edge of the AALP exceeded 12.9 mm (13.8 mm in men,12.7 mm in women), or the anteroposterior diameters of the AALP exceeded the anteroposterior diameters of the acromion by 33.5%.

**Conclusion:**

We could predict the occurrence and development of the related rotator cuff injury in symptomatic patients with specific 3D changes in their acromion and intervene in the acromion of such patients as early as possible to prevent possible rotator cuff injuries in the future.

## Graphical abstract

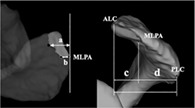

To analyze the relationship between the acromial morphology and the related rotator cuff injury using a 3D measurement technology. We could predict the occurrence and development of the related rotator cuff injury by symptomatic patients with specific 3D changes in their acromion and intervene in the acromion of such patients as early as possible to prevent possible rotator cuff injuries in the future.

## Introduction

Rotator cuff tears (RCT) are a common shoulder disease in adults, occurring in 25% of individuals over 60 years of age and 50% of individuals over 80 years of age^1^. The underlying cause of rotator cuff injuries is still poorly understood. The debate over this issue has been going on since the late 19th century, with the disagreement centering around whether the pathology seen in rotator cuff tears is caused by inherent degeneration of the tendons, contact of the tendons with some structures, or both.

Acromial morphology has been found to be related to rotator cuff injury and glenohumeral arthritis^2^, ^3^. Charles S. Neer II, MD was the first to coin the term “impingement lesions,” which he described as tears of the rotator cuff caused by contact of the rotator cuff with the anterior acromion and the coracoacromial ligament ^4^, ^5^. In 1986, Bigliani classified the acromial morphology into three types: type I was flat, type II was curved, and type III was hooked. Type III was considered to increase the risk of rotator cuff injury ^6^. In 2006, Nyffeler proposed the concept of the acromion index ^7^, with many studies having since shown that an increase in the acromion index significantly increases the risk of rotator cuff injury ^7^, ^8^, ^9^. The critical shoulder angle (CSA) is also widely used to assess the risk of rotator cuff tear and re‐tear after reconstruction. The risk of rotator cuff injury increases when the angle is greater than 35°, while the risk of glenohumeral arthritis increases when the angle is less than 28°^2^, ^10^, ^11^. Most of the studies mentioned above used two‐dimensional measurement based on X‐ray films. Other studies have used a three‐dimensional (3D) model of the scapula to measure and analyze acromial morphology. Using 3D reconstructions, Naidoo *et al*. described the delto‐fulcral triangle, defined by the anterior point of the coracoid process, the most lateral and the most posterior point of the acromion with respect to the scapular plane. The authors found that longer distances between the lateral and posterior acromial aspects and the greater lateral and posterior angles of this triangle are associated with RCT and glenohumeral arthritis ^3^. Li *et al*. used a 3D analysis method to study the relationship between various acromial morphologies and subacromial impingement and demonstrated that some of the acromial morphological parameters were significantly related to subacromial impingement ^12^. A Chinese study measured the anterior spur of the acromion using a 3D model. The study showed that the anterior spur of the acromion was more common in type III acromion and could significantly increase the risk of subacromial impingement and rotator cuff injury ^13^.

In summary, although there are many studies on the relationship between acromial morphology and rotator cuff injury, there is a lack of direct evidence on whether these acromial morphological characteristics can actually cause rotator cuff injuries. To date, it is unknown whether the morphological change in a specific region of acromion will directly impact the rotator cuff tissue and eventually cause tears. Therefore, the current study uses 3D measurement technology to study new 3D acromial morphological characteristics, combined with arthroscopic rotator cuff exploration to find out which acromial morphological characteristics can predict the occurrence of rotator cuff injury, so that clinicians can intervene as early as possible to prevent the inevitable injury of the rotator cuff in the future. We hypothesized that: (i) the morphological change in a specific region of the acromion can directly impact the rotator cuff and cause the tear in the corresponding region; and (ii) the 3D acromial morphology can predict the occurrence and development of the related rotator cuff injury.

## Materials and Methods

### *General Information*

A total of 226 patients (113 men and 113 women) who underwent shoulder arthroscopy from June 2015 to December 2019 at the Department of Orthopedics at our hospital were selected for inclusion in the study. A total of 113 shoulder joints of age‐matched healthy people in the “Development and Application of Internet + Shoulder Arthroscopy Registration System” of our department were selected as the control group. All patients were admitted to the hospital due to pain with limited mobility of the shoulder before surgery. The patients were diagnosed as having subacromial impingement syndrome, subacromial bursitis, and rotator cuff injury by X‐ray, CT scan, and MRI examination before surgery. All patients underwent arthroscopic shoulder surgery.

### *Inclusion and Exclusion Criteria*

Inclusion criteria: (i) patients with type II acromion or type III acromion or shoulder joint adhesion and subacromial bursitis caused by various reasons and rotator cuff tear found by MRI; (ii) patients who underwent arthroscopic repair of the rotator cuff; (iii) acromial morphological characteristics were measured on the 3D model; and (iv) retrospective study.

Exclusion criteria: (i) skeletal malformation of the shoulder; (ii) previous history of trauma, surgery, fractures, infections, and tumors of the shoulder; and (iii) acromioclavicular arthritis and progressive glenohumeral arthritis. All patients fully understood the process and the significance of the study. Informed consent was obtained from all patients.

### *Preoperative Examination*

**Fig. 1:**
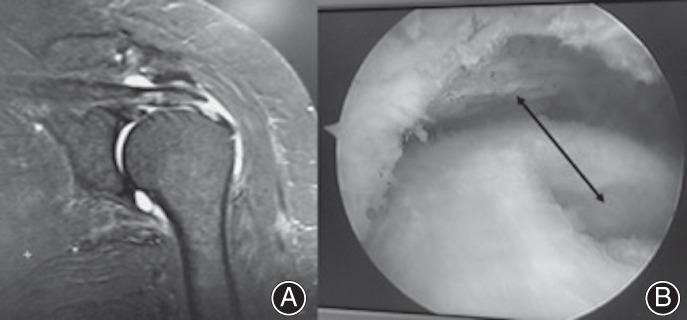
MRI images and findings during surgery. (A) Preoperative MRI images. (B) The corresponding rotator cuff injury in arthroscopy. The arrow showed the correspondence between injury and osseous proliferative structure of acromion.

All the patients showed Neer sign (+), Hawkins sign (+), and Jobe sign (+) in preoperative physical examinations. AUTHOR: All patients were examined using MRI (Fig. 1A), and fulfilled the indications for shoulder arthroscopy.

### *Surgical Procedures*

All the operations were performed by one senior chief physician. The patient was routinely placed in the lateral position. The affected limb was placed on a traction frame for horizontal and vertical traction. The standard posterior and anterior approaches were established to observe the glenohumeral joint. The posterior approach was used to explore the subacromial space, and a standard lateral approach was established. Whether there were any rotator cuff tears and the extent and size of the tear were evaluated after shaving the subacromial bursa (Fig. 1B). Whether the rotator cuff was injured and the extent and size of the injury was determined by the surgeon. The degree of injury was classified as complete rotator cuff injury, partial rotator cuff injury, or no injury. The complete rotator cuff injury was defined as a full‐thickness injury. Ellman classified rotator cuff tears into three categories: bursa‐side tears, inter‐tendon tears, and joint‐side tears. Each category was divided into three degrees according to the tear depth: I degree <3 mm, II degree 3‐6 mm, III degree >6 mm or more than 50% of the thickness of the tendon ^14^. Full‐thickness rotator cuff injury and III‐degree partial rotator cuff injury were routinely performed with rotator cuff repair or suture fixation. A single row of anchor screws was used for fixation. In other cases, no repair or suture would be carried out.

### *Subjects Grouping*

If a rotator cuff tear existed, the surgeon assessed the correspondence between the tear site and the 3D osseous proliferative structure of the acromion after the assistant loosened and abducted the upper limb. If the rotator cuff tear site corresponded to the 3D osseous proliferative structure of the acromion after abduction of the affected limb (Fig. 1B), this kind of tear was considered as a result of bony impact of the acromion. The corresponding patients were classified into the impingement injury group (II group). This group was determined together by a senior chief physician and a junior chief physician. The patients whose rotator cuff tear site did not correspond to the 3D osseous proliferative structure of the acromion were classified into the non‐impingement injury group (NII group). Shoulders of healthy people were included in the control group.

### *CT* *Scan Image Processing and Three‐Dimensional Model Reconstruction*

All subjects underwent CT scans (LightSpeed VCT, GE Healthcare, London, UK) before and after arthroscopic surgery. Image data were acquired with a 130‐kV tube voltage, 512 × 512 acquisition matrix, 0.625‐mm slice thickness, 0.75 pitch, and 170 milliamperage‐seconds baseline tube current. Digital image and communications in medicine (DICOM) image data were imported into Medraw (Image Medraw Technology, Shanghai, China) 3D model reconstruction software. A 3D model of the bony structure with a threshold segmentation range of 180–220 HU was selected. The author used hand‐painted functions to repair bone structures with low HU values, and used construction as well as seed‐filling functions to distinguish humerus, clavicle, and coracoid processes. The reconstructed 3D model for measurement was finally obtained.

### *Establishment of Three‐dimensional Model Coordinate System*

**Fig. 2:**
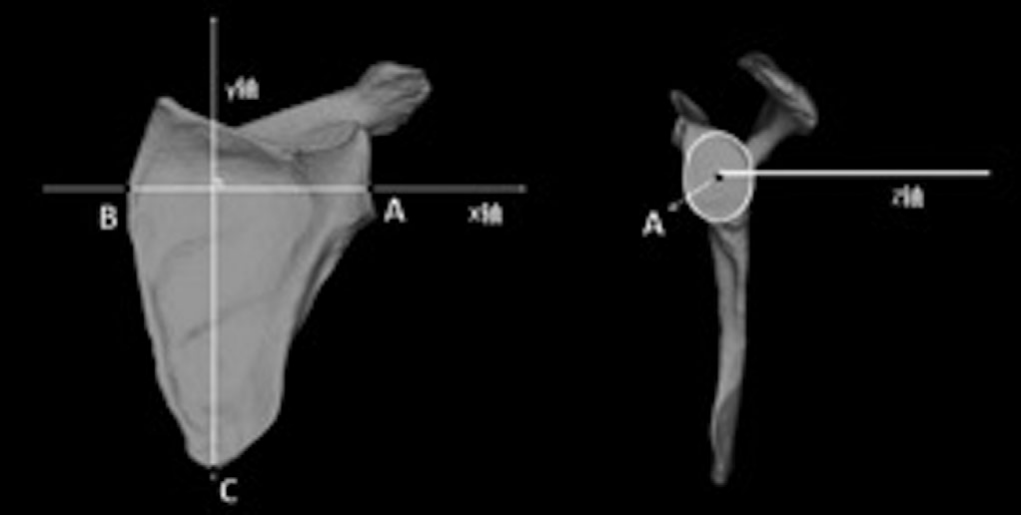
True anteroposterior (AP) view of glenoid and coordinate system. A point: Center of the best‐fit circle of the inferior glenoid. B point: Scapular spine intersects the medial border of the scapula. C point: The most inferior point on the inferior scapular angle.

The center of the best‐fit circle of the inferior glenoid (A point), the B point where the scapular spine intersects the medial border of the scapula, and the most inferior point (C point) on the inferior scapular angle were simulated by a software algorithm. A true AP view of the glenoid was formed by points A, B, and C. A point was used as the origin to form the x‐axis, y‐axis, and z‐axis in the medial–lateral direction, the up–down direction, and the anterior–posterior direction, respectively ^15^ (Fig. 2).

### *Three‐Dimensional* *Morphology Measurement of the Acromion*

#### *Acromiohumeral Interval*

**Fig. 3:**
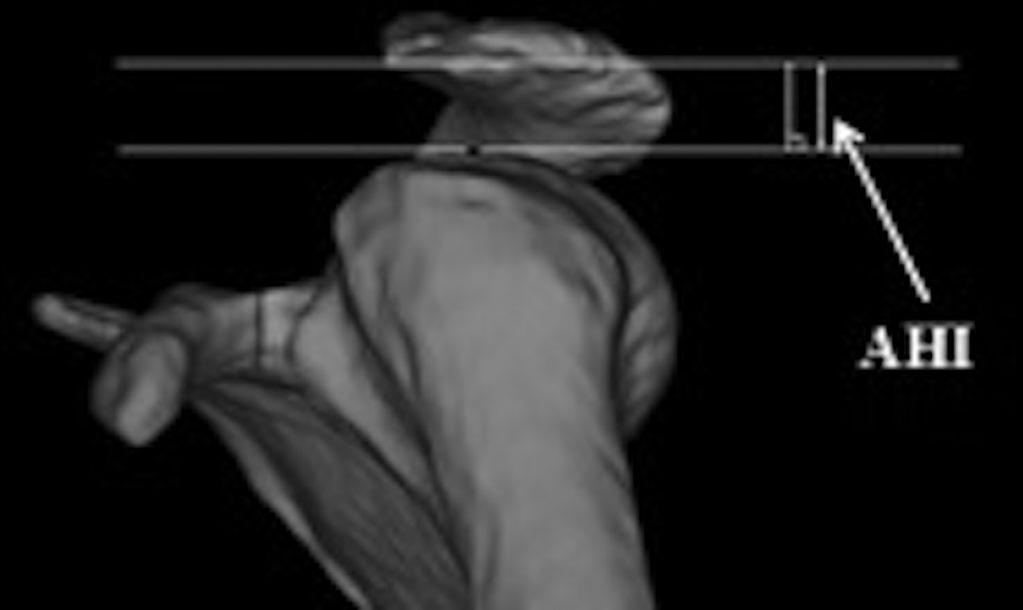
Acromiohumeral interval (AHI) measurement method. Distance between the inferior aspect of the acromion and the most superior point of the humeral head on outlet view image.

Acromiohumeral interval (AHI): Distance between the inferior aspect of the acromion and the most superior point of the humeral head on outlet view image (Fig. 3). The AHI has been proven to be closely related to rotator cuff injury. The risk of rotator cuff injury is increased when the AHI is smaller ^16^.

#### *Acromial Anterior Protrusion and Acromial Inferior Protrusion*

**Fig. 4:**
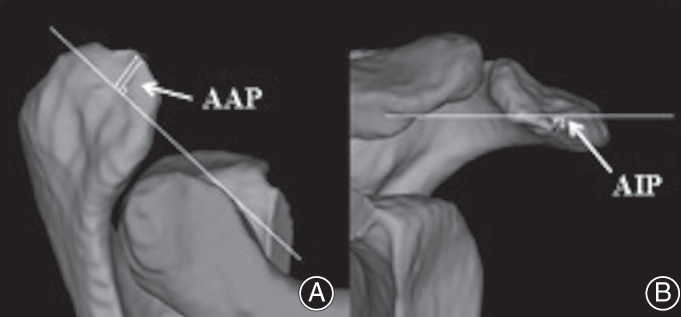
Acromial anterior protrusion (AAP) and acromial inferior protrusion (AIP) measurement method. (A) A line coincident with the anterior aspect of the distal clavicle was drawn on the x–z plane. The distance from the most anterior point of the acromion to this line was the AAP. (B) AIP was defined as the distance from the most inferior point of the anterior acromion to the line which was coincident with the inferior aspect of the distal clavicle on the x–y plane.

Acromial anterior protrusion (AAP): An index used to describe the degree of anterior projection of the acromion (Fig. 4A). A line coincident with the anterior aspect of the distal clavicle was drawn on the x–z plane. The distance from the most anterior point of the acromion to this line was the AAP.

Acromial inferior protrusion (AIP): An index used to describe the degree of anteroinferior projection of the acromion (Fig. 4B). AIP was defined as the distance from the most inferior point of the anterior acromion to the line which was coincident with the inferior aspect of the distal clavicle on the x–y plane.

The above two indices were first proposed by Li *et al*. in 2017 indices^12^.

#### *Acromioclavicular Angle*

**Fig. 5:**
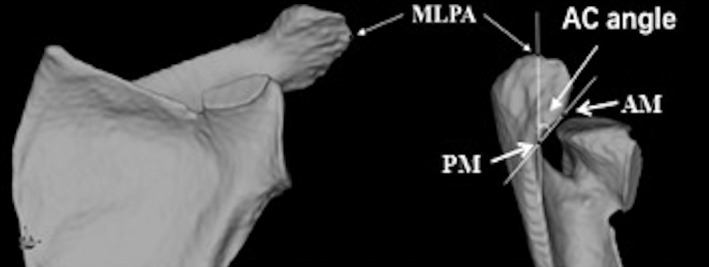
The acromioclavicular angle (AC) measurement method. Most lateral point of acromion (MLPA) was determined on the x–y plane. The AC angle was defined as the angle between the anterior joint line margin (AM), the MLPA, and the posterior joint line margin (PM).

Acromioclavicular angle (AC angle): The AC angle was used to describe the relationship between the most lateral point of the acromion (MLPA) and the acromioclavicular joint (Fig. 5). It can reflect the proportion of the acromion area anterior to the MLPA of the entire acromion ^15^.

#### *Anatomy of Acromial Anterolateral Protrusion*

**Fig. 6:**
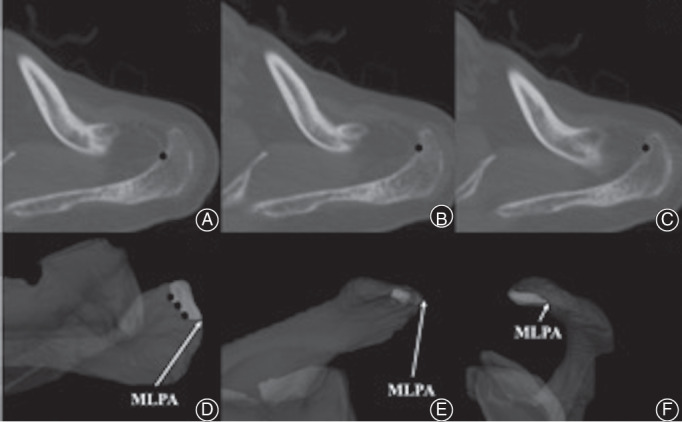
Localization of acromial anterolateral protrusion (AALP). (A), (B), (C) The inflection point (black dots) with the greatest curvature of the anterolateral curve was marked along the inner edge of the acromion on the CT image of the anterior half of the acromion. (D) Position of the point marked on the CT image in the x–z plane of the three‐dimensional model. The edge formed by the black dots were the inner edge of the AALP (dark white area). (E) x–y plane view of AALP. (F) y–z plane view of AALP. MLPA, most lateral point of acromion.

The current study proposed a new 3D morphological method to assess the acromion. In the 3D acromion model, the acromion was divided into the anterior half and the posterior half, with the MLPA point as the boundary. The anatomic structure of the anterior half of the acromion was considered to be a risk factor for the rotator cuff injury caused by the corresponding impact of the acromion. The inflection point with the greatest curvature of the anterolateral curve was marked along the inner edge of the acromion on the CT image of the anterior half of the acromion. The corresponding inflection points on the 3D model were simulated by computer to obtain the inner edge of the acromial anterolateral protrusion (AALP) (Fig. 6). To some extent, the size and range of the AALP determined the risk of rotator cuff injury. The morphology of AALP could also provide a reference for the range of acromioplasty in arthroscopy.

**Fig. 7:**
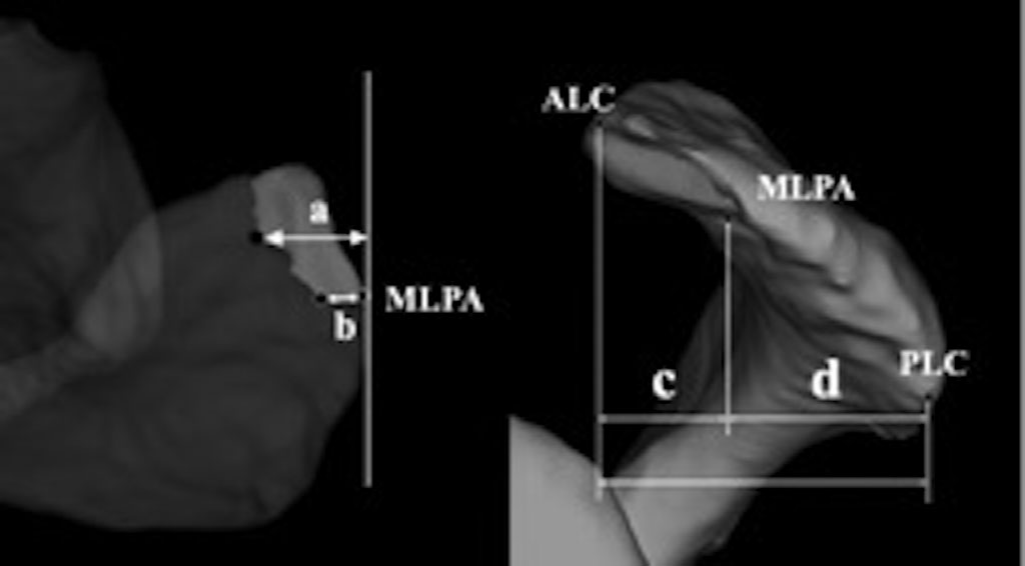
Acromial anterolateral protrusion (AALP) measurement method. (a) The distance from the most medial edge of the AALP to the most lateral point of the acromion (MLPA). (b) The distance from the most posteromedial edge of the AALP to the MLPA. (c) Anteroposterior diameters of the AALP. (c + d) Anteroposterior diameters of the acromion. (c/c + d) × 100(%): The proportion of anteroposterior diameters of AALP to the anteroposterior diameters of acromion. Anterolateral corner of acromion; PLC, posterolateral corner of the acromion.

The distance from the most medial edge and the posteromedial edge of the AALP to the MLPA was measured on the x–z plane. The anteroposterior diameters of the AALP and the proportion of anteroposterior diameters of the AALP to the anteroposterior diameters of the acromion were measured on the y–z plane (Fig. 7). The distance from the most medial edge of the AALP to the MLPA was defined as (a). The distance from the most posteromedial edge of the AALP to the MLPA was defined as (b). (c) represented the anteroposterior diameters of AALP. (c + d) represented the anteroposterior diameters of the acromion. (c/c + d) × 100(%) represented the proportion of the anteroposterior diameters of AALP to the anteroposterior/diameters of the acromion.

### *Statistical Analysis*

The SPSS (Version 20.0, IBM, USA) was used for statistical processing. The coefficient of variation (CV%) and the intragroup correlation coefficient (ICC%) between measurements of various morphological parameters of the acromion were used to verify the reliability and the consistency of the measurement. CV% < 5% indicates very good reliability. ICC% > 75% shows that variability between duplicates is not a source of any significant variation. The Shapiro–Wilk test was used to verify the normal distribution of the parameters. One‐way analysis of variance was used to compare the age and the 3D morphological parameters of the acromion among the control group, the NII group and the II group. *P* < 0.05 was considered statistically significant.

## Results

Measurements were performed independently by two junior physicians. Physicians were completely unaware of the details of the patients. The average of the two physicians' measurement results was used for statistical analysis.

### *General Results*

Table 1 shows the number, mean age, and sex for the three groups. The average age of the control group, the NII group, and the II group was 60.3, 59.3, and 62.3 years, respectively. There was no statistically significant difference in age (*P* = 0.8416) among the three groups.

**TABLE 1:** General information of patients

Group	Control group	NII group	II group	*P*‐value
n	113	98	128	—
Age	60.3 ± 7.1	59.3 ± 4.8	62.3 ± 8.2	0.8416
Sex	56/57	49/49	64/64	—

II, impingement injury group; NII, non‐impingement injury group. Age (years, $\bar{x}$ ± s); sex (*n*, male/female); *P*, intergroup difference, <0.05 indicates statistical significance.

The results of the coefficient of variation and the intragroup correlation coefficient are shown in Table 2. The results of the intraobserver (<5%) and interobserver variability (>87%) analysis revealed the parameters to have high intraobserver and interobserver concordance.

**TABLE 2:** Coefficient of variation and intragroup correlation coefficient between measurements of various morphological parameters of acromion

Parameters	Control group	NII group	II group	CV (%)	ICC (%)
AHI (mm)	8.6	8.4	8.1	3.5	92.8
AAP (mm)	6.8	6.7	8.5	1.5	97.3
AIP (mm)	8.5	8.9	9.0	2.3	87.7
AC angle (°)	41.2	40.8	40.8	1.0	91.5
(a) (mm)	12.9	13.8	14.8	1.3	97.4
(b) (mm)	9.7	10.1	11.0	3.7	97.4
(c) (mm)	10.0	10.3	11.3	1.2	95.3
(c/c + d) × 100 (%)	28.1	29.0	31.6	2.7	96.5

Parameters ($\bar{x}$). II, impingement injury group; NII, non‐impingement injury group. Coefficient of variation (CV%): The random differences between replicates as a % of the mean (<5% indicates very good reliability). The intragroup correlation coefficient (ICC%) represents the variability between patients as a % of the total variation among readings. Values close to 100% indicate that variability between duplicates is not a source of any significant variation.

AAP, acromial anterior protrusion; AC angle, acromioclavicular angle; AHI, acromiohumeral interval; AIP, acromial inferior protrusion.

### *Acromial Morphological Distance Parameters*

Table 3 showed the average value, the standard deviation, the 95% confidence interval (CI), and the statistical analysis results of the parameters in three groups.

**TABLE 3:** Differences in acromial morphological parameters among the three groups

Parameters	Control group		NII group		II group		*P*‐value
	Mean ± SD 95%CI		Mean ± SD 95%CI		Mean ± SD 95%CI		
AHI (mm)	8.6 ± 1.7	7.4–9.7	8.4 ± 0.8	7.2–9.6	8.1 ± 2.0	6.8–9.4	0.0540
AAP (mm)	6.8 ± 3.5	4.5–9.1	6.7 ± 3.4	3.4–10.1	8.5 ± 4.7	5.6–11.5	0.0374
AIP (mm)	8.5 ± 5.7	6.9–10.1	8.9 ± 2.7	6.6–11.3	9.0 ± 3.1	7.0–11.0	0.4197
AC angle (°)	41.2 ± 4.5	38.2–44.3	40.8 ± 3.8	37.7–43.8	40.8 ± 2.7	39.1–42.5	0.9603
(a) (mm)	12.9 ± 2.1	11.0–14.8	13.8 ± 1.5	12.1–15.4	14.8 ± 1.7	12.9–16.8	0.0176
(b) (mm)	9.7 ± 2.8	7.8–11.5	10.1 ± 1.0	8.6–11.6	11.0 ± 2.0	9.1–12.9	0.0018
(c) (mm)	10.0 ± 3.2	7.9–12.1	10.3 ± 1.5	8.0–12.7	11.3 ± 3.2	8.6–13.9	0.0361
(c/c + d) × 100 (%)	28.1 ± 2.8	26.9–29.3	29.0 ± 2.4	27.5–30.5	31.6 ± 3.0	29.7–33.5	0.0383

AAP, acromial anterior protrusion; AC angle, acromioclavicular angle; AHI, acromiohumeral interval; AIP, acromial inferior protrusion; CI, confidence interval; II, impingement injury group; NII, non‐impingement injury group; *P*, intergroup difference in acromial morphological parameters (<0.05 indicates statistical significance); SD, standard deviation.

#### *Acromiohumeral Interval*

The AHI in the II group, the NII group, and the control group were 8.1, 8.4, and 8.6 mm, respectively. The values of AHI in the II group were reduced by 3.8% and 5.8%, respectively, compared with the NII group and the control group. However, the differences among the three groups were not statistically significant; *P* = 0.054.

#### *Acromial Anterior Protrusion*

**Fig. 8:**
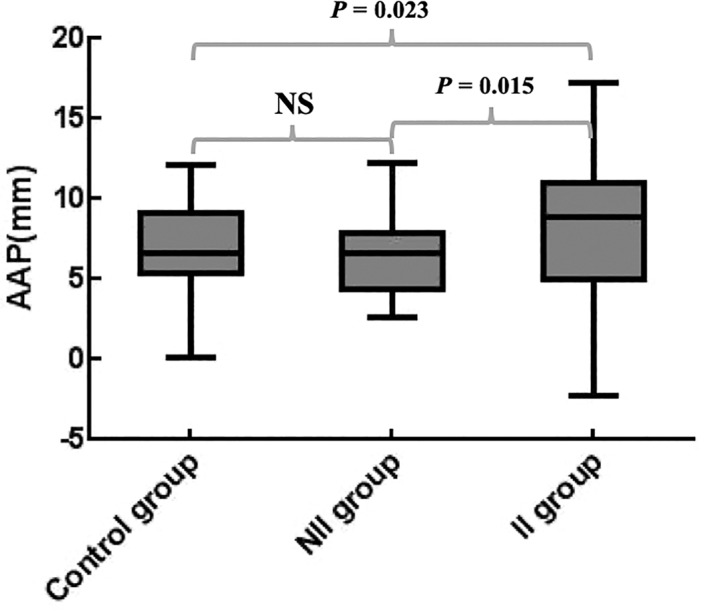
Box diagram of the differences in acromial anterior protrusion (AAP) among the three groups. NS, not significant.

However, the AAP among the three groups showed significant differences; *P* = 0.0374. The AAP in the II group, the NII group, and the control group were 8.5, 6.7, and 6.8 mm, respectively. The values of AAP in the II group were increased by 26.9% (*P* = 0.015) and 25% (*P* = 0.023), respectively, compared with the NII group and the control group (Fig. 8). The AAP increased significantly in the II group. The 95% CI of the AAP in the II group was 5.6–11.5 mm.

#### *Acromial Inferior Protrusion*

The AIP in the II group, the NII group, and the control group were 9.0, 8.9, and 8.5 mm, respectively. The values for AIP in the II group were increased by 1.1% and 5.9%, respectively, compared with the NII group and the control group. The differences among the three groups were not statistically significant; *P* = 0.4197.

### *Acromial Morphological Angle Parameters*

The values of the AC angle in the three groups were close. The AC angle in the II group, the NII group, and the control group were 40.8°, 40.8°, and 41.2°, respectively. The values of the AC angle in the II group were reduced by 0% and 1%, respectively, compared with the NII group and the control group. The differences among the three groups were not statistically significant; *P* = 0.9603 (Table 3).

### *Parameters of Acromial Anterolateral Protrusion*

#### *Distance from the Most Medial Edge of the Acromial Anterolateral Protrusion to the Most Lateral Point of the Acromion (a)*

**Fig. 9:**
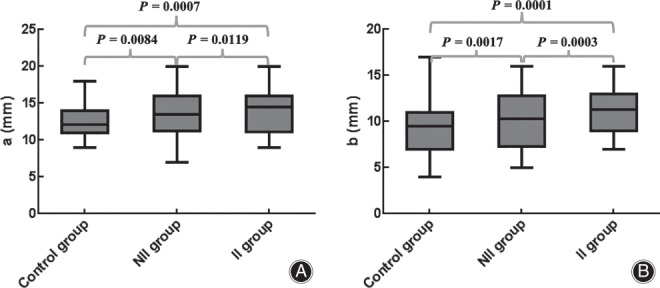
Box diagram of the differences of (a) and (b) among the three groups. (A) Differences of (a) among the three groups. (B) Differences of (b) among the three groups.

The (a) increased gradually among the control group, the NII group, and the II group. (a) among the three groups showed significant differences; *P* = 0.0176. (a) in the II group, the NII group, and the control group were 14.8, 13.8, and 12.9 mm, respectively. The values of (a) in the II group were increased by 7.2% (*P* = 0.0119) and 14.7% (*P* = 0.0007), respectively, compared with the NII group and the control group (Fig. 9A). The values of (a) in the NII group were also increased by 7% (*P* = 0.0084) compared with the control group. The results showed that (a) in patients with rotator cuff injuries was larger than in the normal population. The 95% CI of (a) in the II group and the NII group were 12.9–16.8 mm and 12.1–15.4 mm, respectively.

#### *Distance from the Most Posteromedial Edge of the Acromial Anterolateral Protrusion to the Most Lateral Point of the Acromion (b)*

The (b) also increased gradually among the control group, the NII group, and the II group. (b) among the three groups showed significant differences; *P* = 0.0018. (b) in the II group, the NII group, and the control group were 11.0, 10.1, and 9.7 mm, respectively. The values of (b) in the II group were increased by 9% (*P* = 0.0003) and 13.4% (*P* = 0.0001), respectively, compared with the NII group and the control group (Fig. 9B). The values of (b) in the NII group were also increased by 4.1% (*P* = 0.0017) compared with the control group. The same as for (a), (b) in patients with rotator cuff injuries was larger than in the normal population. The 95% CI of (b) in the II group and the NII group were 9.1–12.9 mm and 8.6–11.6 mm, respectively.

#### *Anteroposterior Diameters of Acromial Anterolateral Protrusion (c)*

The (c) among the three groups showed significant differences; *P* = 0.0361. (c) in the II group, the NII group, and the control group were 11.3, 10.3, and 10.0 mm, respectively. The values of (c) in the II group were increased by 9.7% and 13%, respectively, compared with the NII group and the control group. (c) increased significantly in the II group. The 95% CI of (c) in the II group was 8.6–13.9 mm.

**Fig. 10:**
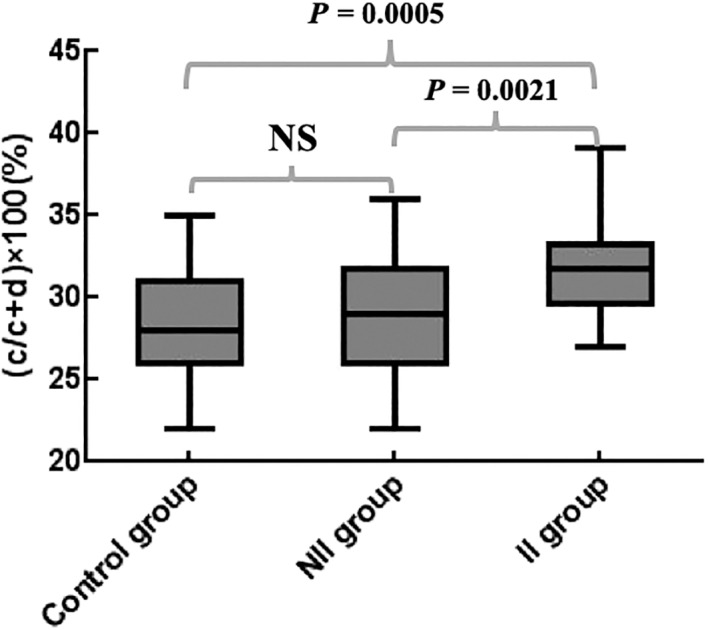
B*ox diagram of the differences of (c/c + d) × 100 (%) among the three groups. NS, not significant.*

The proportion of anteroposterior diameters of AALP to the anteroposterior diameters of acromion [(c/c + d) × 100(%)] among the three groups showed significant differences; *P* = 0.0383. [(c/c + d) × 100(%)] in the II group, the NII group, and the control group were 31.6%, 29.0% and 28.1%, respectively. The values of [(c/c + d) × 100(%)] in the II group were increased by 8.9% (*P* = 0.0021) and 12.5% (*P* = 0.0005), respectively, compared with the NII group and the control group (Fig. 10). The anteroposterior size of the AALP in the II group was significantly larger than in the other two groups. The 95% CI of [(c/c + d) × 100(%)] in the II group was 29.7%–33.5%.

## Discussion

### *Impingement Syndrome Theory* Versus *Intrinsic “Fatigue‐Failure” Theory*

Rotator cuff disease (RCD) is one of the main causes of shoulder pain and dysfunction. Once it occurs, it seriously affects the daily life of patients. The debate over the cause of rotator cuff disease and its treatment has been around since the late 19th century, with the disagreement centering around whether the pathology seen in rotator cuff tears is caused by inherent degeneration of the tendons, contact of the tendons with some structures, or both. The theory of degeneration supports the view that most rotator cuff defects are the result of age‐related degeneration in the quality of the tendon. With the increase in patients' age, the synovium around the rotator cuff undergoes inflammatory changes, and the arrangement of tendon fibers at the attachment of rotator cuff is disordered, resulting in cell degeneration, metatropy, and necrosis, which leads to the decreased compliance of rotator cuff tendons, eventually causing RCD ^17^, ^18^, ^19^. However, the theory of impingement supports the idea that osteoarthritis occurs in the bony structure of the acromion, and then different forms of subacromial osteophytes are formed, which can reduce the subacromial space, and directly stimulate the subacromial synovium and rotator cuff tissue to cause the tear during activity ^20^. In 1972, Dr Neer reported a “characteristic ridge of proliferative spurs and excrescences on the undersurface of the anterior process (of the acromion) apparently caused by repeated impingement of the rotator cuff and the humeral head, with traction of the coracoacromial ligament” ^4^. The morphology of the acromion varies greatly from individual to individual.

At present, there is a lack of evidence to study the morphological structure of acromion, which can cause the corresponding rotator cuff injury *in vivo*. Hamid ^21^ and Toivonen ^22^ reported that patients with rotator cuff injuries had a larger anterolateral osseous proliferative structure of the acromion. Yamamoto revealed that changes in the anatomic morphology of the acromion may reduce the incidence of rotator cuff degenerative tear and re‐tear after repair ^23^. Therefore, when we discover specific morphological characteristics of acromion in patients with rotator cuff injuries, we might ask: Is the rotator cuff lesion leading to a change in the configuration of the acromial morphology or a change in the bony structure in a specific region of the acromion leading to the rotator cuff injury in the corresponding region? Or are they both products of genetics and aging? When rotator cuff injury was explored in shoulder arthroscopy, the protrusion structure of bone hyperplasia could also be observed in the corresponding region of the acromion, and the impact between the anterolateral osseous proliferative structure of the acromion and the injured rotator cuff could occur on the position of upper limb abduction. According to the latest literature review on the relationship between acromial morphology and rotator cuff injury, type III acromion and an increase in the acromion index could significantly increase the risk of non‐traumatic rotator cuff injury, but no evidence was found to support the view that rotator cuff injury was caused by the impact of the corresponding region of the acromion ^24^, ^25^.

Based on previous research, the present study analyzed the interrelationship between the 3D morphology of the acromion and rotator cuff injuries in patients with non‐traumatic shoulder pain undergoing arthroscopic surgery. We sought to find evidence of rotator cuff injury caused by acromion impact, and to predict the occurrence and development of rotator cuff injury by symptomatic patients with specific 3D changes in the acromion. The results could guide clinicians to intervene and treat RCD as early as possible to prevent or avoid the occurrence and development of rotator cuff injuries in the future.

### *Three‐Dimensional Measurement Advantages*

The lateral scapula, outlet view X‐ray, and MRI images are still the most commonly used methods for the study of acromion morphology. Although some researchers have improved the shooting angle of outlet view X‐ray film to show the acromion structure to the greatest extent, it is still difficult to obtain a uniform standard outlet view X‐ray film ^26^. Therefore, the 3D model of the acromion based on CT images can effectively solve this problem. The present study referred to the 3D coordinate system based on the morphology of the scapula established by Jacxsens in 2016 ^27^. On the plane formed by the three points of the center of the best‐fit circle of the inferior glenoid, the scapular spine intersects the medial border of the scapula and the most inferior point on the inferior scapular angle was used as the true AP view of the glenoid. Based on this plane, the position of the 3D model could be adjusted in a standardized and quantitative way, which made the observation of the 3D model more comprehensive and the measurement more accurate ^28^.

### *Effect of the Three‐Dimensional Morphology of the Acromion on the Related Rotator Cuff Injury*

In the current study, the relationship between the 3D morphology of the acromion and the rotator cuff injury observed during arthroscopy was analyzed in groups for the first time. There was no significant difference in age among the three groups; *P* = 0.8416. Some studies show that the acromial anterolateral protrusion increases with age ^29^.Other studies suggest that changes in acromial morphology are independent of age ^8^, ^30^. Even though there was a certain correlation between the acromial anterolateral protrusion and age, the results of this study showed that there was no significant relationship between age and whether these structures could impact the rotator cuff tissue and cause injury.

The AHI is an important indicator of the degree of superior displacement of the humeral head. In huge rotator cuff injury, superior capsule relaxation and biceps long head tendon, the superior displacement of the humeral head increased; in contrast, the AHI decreased ^31^, ^32^, ^33^. The evaluation of AHI was mostly measured on X‐rays and MRI. Recent research by Mirzayan showed that the measurement results of the AHI on X‐rays were larger than those on MRI and decreased with the increased risk of rotator cuff injury. However, the AHI in their study was evaluated on the AP view of the shoulder, which was different from our study ^34^. Li *et al*. performed a 3D measurement and evaluation of AHI. The results showed that the average AHI in the impact group was 5.5 mm, and in the control group was 6.5 mm. The difference was statistically significant ^12^. Hai‐Peng Liu used 3D reconstruction technology to measure the AHI in a 3D model to evaluate the efficacy of subacromial decompression in the treatment of acromial impingement syndrome. The results showed that the average AHI on the affected side was 5.4 mm ^35^. In the current study, the average values of AHI in the control group, the NII group, and the II group were 8.6 mm, 8.4 mm, and 8.1 mm respectively, which were larger than the results of the study by Li *et al*. The differences might be related to the position difference of the 3D model during measurement. The AHI value of both men and women in the current study gradually decreased in the control group, the NII group and the II group, and the AHI value of the II group was the smallest, but there was no significant difference among the three groups; *P* = 0.054. The AHI might be related to the degree of the rotator cuff injury, but it did not reflect the possible impact between the acromial anterolateral protrusion and the rotator cuff tissue.

The AAP and the AIP were the indicators reflecting the relationship between the acromion and the clavicle. The AAP represented the anterior part of the acromion that extended beyond the clavicle, which was usually embedded in the coracoacromial ligament and coracoacromial arch. The AIP represented the inferior part of the acromion that extended beyond the clavicle, which was usually the target area of acromioplasty. Injuries at the anteromedial site of the rotator cuff were often associated with these two parameters. The present study showed that there were significant differences in the AAP among the three groups. The results were the same for men and women. The control group and the NII group did not show differences, while the AAP in the II group was significantly larger than in the control and NII groups; *P* = 0.023 and 0.015, respectively. The increase of AAP might cause the AALP to collide with the rotator cuff tissue at the corresponding site, which could predict the occurrence and development of the related rotator cuff injury. The AIP did not show significant differences among the three groups; *P* = 0.4197. The above results were the same for men and women, respectively. The results of Li *et al*. showed that the AAP and the AIP in the impact group were significantly larger than those in the normal group, and some of them were similar to our results ^12^. This might be related to the large variation of the tangent to the subclavian surface during AIP measurement. The ICC% value of the AIP in this study was only 87.7%, and the bias of measurement might be relatively large. Whether AIP can be used as a reliable evaluation indicator remains to be verified.

The AC angle was an index reflecting the position of the MLPA relative to the acromioclavicular joint line. The larger the AC angle was, the more acromial bony structure existed in front of the MLPA. The more anterior acromial bony structure existed, the higher the risk of subarcomial impingement and rotator cuff wear during activity. In this study, the average AC angle was between 37° and 44°, which was approximately 8° smaller than the 49° reported by Karns ^15^.There was no significant difference in the AC angle among the three groups; *P* = 0.9603. The above results were the same for men and women, respectively. The AC angle only reflected the area of the acromion in front of the MLPA, and it did not reflect the risk of the AALP structure colliding with the rotator cuff tissue.

### *Relationship of Acromial Anterolateral Protrusion and Rotator Cuff Injury*

The most important finding in this study was to propose a new method for 3D morphological assessment of the acromion. The size and range of the AALP were measured three‐dimensionally with MLPA as the boundary. The MLPA was better recognized in the 3D model of the acromion. It also served as a reference point for the acromion index, the CSA angle, and the range of acromioplasty ^10^, ^36^. In the present study, the distance from the most medial and posteromedial edge of the AALP to the MLPA represented the maximum width of the medial–lateral (a) and lateral (b) edges of the AALP. The average values of (a) and (b) in the control group, the NII group, and the II group gradually increased, and the differences were statistically significant. The *P‐*values were 0.0176 and 0.0018, respectively. The above results were the same for men and women, respectively. The AALP was wider in patients with rotator cuff injuries. The (a) and (b) of the II group were significantly larger than those of the NII group; *P‐*values were 0.0119 and 0.0003, respectively. The (a) and (b) in patients with rotator cuff injuries were larger than in the normal population; *P* < 0.05. This suggested that the larger width of AALP might cause the related rotator cuff injury. In the II group, the 95% CI of (a) was 12.9–16.8 mm (13.5–17.4 mm in men and 10.8–15.1 mm in women) and the 95% CI of (b) was 9.1–12.9 mm (9.9–13.8 mm in men and 8.9–12.7 mm in women). Therefore, clinicians should pay special attention to these two parameters when evaluating the 3D morphology of the anterior and lateral sides of the acromion. The risk of rotator cuff injury due to subacromial impingement was increased when the maximum width of the medialolateral edge of the AALP exceeded 16.8 mm (17.4 mm in men and 15.1 mm in women) or the maximum width of posterior edge of the AALP exceeded 12.9 mm (13.8 mm in men and 12.7 mm in women). At the same time, these values could also be used as a reference for the range of acromioplasty in shoulder arthroscopy. The proportion of anteroposterior diameters of the AALP to the anteroposterior diameters of the acromion (c/c + d) in this study also showed significant differences among the three groups. The (c/c + d) in the II group was significantly larger than those in the control and the NII groups, with *P‐v*alues of 0.0005 and 0.0021, respectively. The 95% CI of (c/c + d) × 100(%) was 29.7%–33.5%. The risk of rotator cuff injury due to subacromial impingement was also increased when the anteroposterior diameters of the AALP exceeded the anteroposterior diameters of the acromion by 33.5%. Fujisawa measured the acromial morphology through the 3D model of the scapula and revealed that there was a significant correlation between the full‐thickness rotator cuff injury and the bony structure of the acromial hyperplasia extending 2 mm forward or 3 mm outward ^37^. However, Fujisawa's approach was inconsistent with the measurement method of this study. Fujisawa measured the size of the acromial anterolateral protrusion by overlapping the 3D model of the affected side with the healthy side. The 3D measurement in the current study was combined with whether the rotator cuff injury was related to subacromial impingement during the operation, which was more suitable for clinical evaluation and practice.

### *Limitations*

Several limitations exist in the study. First, although whether the rotator cuff injury during surgery is caused by the impact of the corresponding acromial anterolateral protrusion during activity is jointly confirmed by two chief physicians, deviations in judgment may still exist. At present, there is some motion analysis equipment available to simulate the impact test of the shoulder *in vitr*o; however, whether it can be applied to the shoulder *in vivo* to study the mechanism of subacromial impingement syndrome more accurately needs further research. Second, the sample size may have been insufficient to accurately assess changes in the inferior surface and anterior or medial edge of the acromion. Limited by the annual amount of shoulder arthroscopy surgery in our department, a larger sample size is needed to reach a more accurate conclusion. Third, the current study was a cross‐sectional study which could only describe the acromial morphology at a specific time point. A longitudinal observational study would be ideal to confirm the causal relationship between the acromial shape and RCD.

### *Conclusion*

The current study proposed a new method for 3D morphological assessment of the acromion. The morphological change in a specific region of the acromion could directly impact the rotator cuff and cause the tear in the corresponding region. The increase in the AAP might cause the AALP to collide with the rotator cuff tissue at the corresponding site, which could predict the occurrence and development of the related rotator cuff injury. The risk of rotator cuff injury due to subacromial impingement was increased when the maximum width of the medialolateral edge of the AALP exceeded 16.8 mm (17.4 mm in men and 15.1 mm in women), the maximum width of the posterior edge of the AALP exceeded 12.9 mm (13.8 mm in men and 12.7 mm in women), or the anteroposterior diameters of the AALP exceeded the anteroposterior diameters of the acromion by 33.5%. We could predict the occurrence and development of the related rotator cuff injury in symptomatic patients with specific 3D changes in the acromion. The results could guide clinicians to intervene and treat RCD as early as possible to prevent the occurrence and development of rotator cuff injuries in the future. The values of acromial morphological parameters could also be used as a reference for the range of acromioplasty in shoulder arthroscopy.
